# Editorial on Special Issue “Design and Optimization of Pharmaceutical Gels”

**DOI:** 10.3390/gels10010038

**Published:** 2024-01-01

**Authors:** Xuejuan Zhang, Ying Huang, Zhengwei Huang

**Affiliations:** 1 State Key Laboratory of Bioactive Molecules and Druggability Assessment, Jinan University, Guangzhou 511443, China; zhangxj02230223@jnu.edu.cn (X.Z.); huangy2007@jnu.edu.cn (Y.H.); 2 College of Pharmacy, Jinan University, Guangzhou 511443, China

## 1. Introduction

The efficacy of many bioactive agents, including drugs, food supplements, and vaccines, is limited because of their poor chemical stability, low water solubility, and low oral bioavailability [1]. For this reason, delivery vehicles are being developed to overcome these problems [2]. In particular, gels have attracted significant attention in the fields of drug delivery systems (DDSs) [3], such as sustained-release [4], controlled-release [5], targeted [6], and local [7] DDSs, due to their high drug loading efficiency, high biocompatibility, and low toxicity.

Gels are three-dimensional, semi-solid systems consisting of polymeric matrices [8]. The physicochemical properties of gels, such as their physical strength, viscosity, and self-healing ability, can be altered to meet the specific requirements of applications in various fields [9], such as drug and cell delivery [10], bioscaffolds [11], and the modeling of extracellular matrices [12]. In particular, novel gel-based delivery systems (such as intelligent hydrogels, in situ gels, emulsion gels, nanogels, vesicular gels, and microgels) that have emerged in recent years can release drugs via specific biological or external stimuli [13], such as temperature, pH, enzymes, ultrasound, antigens, etc., to achieve precise and local drug delivery [14]. Therefore, gels have broad clinical application prospects, and they are anticipated to provide new, effective, and robust strategies for the theranostics of diseases [15].

In this context, this Special Issue, entitled “Design and Optimization of Pharmaceutical Gels”, in *Gels*, has been established to shed light on gels in terms of material development, system construction, structural characterization, and the effect for disease treatment, and research on gels with high translational potential is particularly sought after. We received submissions from around the world, reflecting high cultural diversity. In addition to this Editorial, this Special Issue comprises 12 items in total, including 9 articles and 3 reviews. It is encouraging that these contributions can boost the development of pharmaceutical gels, and the following content will provide a brief overview of them. It is important to note that the topics of contributions are roughly divided into three categories: hydrogels, nanogels, and gel reviews.

## 2. Overview of Contributions to Hydrogels

Hydrogels are widely used pharmaceutical gel platforms. In this Special Issue, four articles on hydrogels are included, which are briefly introduced as follows:

(1) An article from Korea, entitled “Development of Efficient Sodium Alginate/Polysuccinimide-Based Hydrogels as Biodegradable Acetaminophen Delivery Systems”. In this article, a pharmaceutical sodium alginate/polysuccinimide-based hydrogel platform was developed for acetaminophen delivery, demonstrating good biodegradability and mechanical properties. Systemic characterizations were performed, such as solid-state properties, rheological properties, degradation profile, drug loading and release, cytotoxicity, etc. This platform may have promising application aspects in pain and fever management.
(2) A collaborative article from India, Egypt, and Saudi Arabia, entitled “Piperine-Loaded In Situ Gel: Formulation, In Vitro Characterization, and Clinical Evaluation against Periodontitis”. In this article, a pharmaceutical in situ gel platform was developed for piperine delivery, exhibiting sol-to-gel transformation behavior. Systemic characterizations were performed, such as drug–excipient compatibility, gelling profile, pH, viscosity, syringeability, solid-state properties, drug loading and release, etc. It is noteworthy that a randomized clinical trial was conducted for the designed formulation. This platform may have promising application aspects in periodontitis management.
(3) An article from Thailand, entitled “Response Surface Methodology for Optimization of Hydrogel-Forming Microneedles as Rapid and Efficient Transdermal Microsampling Tools”. In this article, a pharmaceutical hydrogel-forming microneedle platform was developed for dermal interstitial fluid sampling, demonstrating interstitial fluid extraction effects. After utilizing Box–Behnken and central composite designs for the formulations, systemic characterizations were performed, such as morphology, swelling profile, mechanical strength, skin insertion, interstitial fluid extraction and recovery, etc. This platform may have promising application aspects in point-of-care testing.
(4) A collaborative article from Korea and India, entitled “pH Sensitive Drug Delivery Behavior of Palmyra Palm Kernel Hydrogel of Chemotherapeutic Agent”. In this article, a pharmaceutical palmyra palm kernel hydrogel platform was developed for 5-fluorouracil delivery, exhibiting pH-responsive swelling capacities. Systemic characterizations were performed, such as swelling profile, drug loading and release, solid-state properties, etc. This platform may have promising application aspects in colon cancer management.

## 3. Overview of Contributions to Nanogels

Nanogels are defined as pharmaceutical gel platforms with nanometer sizes or those encapsulating nanoparticles. In recent years, nanogels have gained significant research interest. In this Special Issue, five articles on nanogels are included, which are briefly introduced below:

(1) A collaborative article from India and Saudi Arabia, entitled “Design and Development of a Topical Nanogel Formulation Comprising of an Unani Medicinal Agent for the Management of Pain”. In this article, a pharmaceutical nanogel platform was developed for *Matricaria chamomilla* oil delivery, demonstrating enhancements in pain threshold and reductions in skin irritation capacities. Systemic characterizations were performed, such as pH, viscosity, spreadability, extrudability, texture, drug release, etc. This platform may have promising application aspects in migraine management.
(2) An article from Malaysia, entitled “Enhanced Osteogenesis Potential of MG-63 Cells through Sustained Delivery of VEGF via Liposomal Hydrogel”. In this article, a pharmaceutical liposomal hydrogel platform was developed for vascular endothelial growth factor delivery, demonstrating elevated osteogenesis potential. Systemic characterizations were performed, such as morphology, porosity, solid-state properties, drug release, cell–hydrogel co-culture profile, etc. This platform may have promising application aspects in bone regeneration.
(3) A collaborative article from Saudi Arabia and Egypt, entitled “Numerical Optimization of Prednisolone-Tacrolimus Loaded Ultraflexible Transethosomes for Transdermal Delivery Enhancement; Box–Behnken Design, Evaluation, Optimization, and Pharmacokinetic Study”. In this article, a pharmaceutical transethosomal gel platform was developed for prednisolone–tacrolimus co-delivery, exhibiting potential anti-inflammatory effects. After utilizing a Box–Behnken design for the formulations, systemic characterizations were performed, such as drug loading and release, particle size, pH, spreadability, skin permeation, in vivo anti-inflammatory ability and pharmacokinetics, etc. This platform may have promising application aspects in the management of skin inflammatory conditions.
(4) An article from Poland, entitled “Emulsion-Based Gel Loaded with Ibuprofen and Its Derivatives”. In this article, a pharmaceutical emulsion-based gel platform was developed for ibuprofen and derivatives thereof, demonstrating enhanced skin penetration capabilities. It is worth mentioning that the authors synthesized several derivatives of ibuprofen. Systemic characterizations were performed for the gel platform, such as morphology, stability, density, refractive index, viscosity, particle size, skin permeation, etc. This platform may have promising application aspects in the management of inflammatory skin diseases.
(5) An article from China, entitled “Design and Evaluation of Paeonol-Loaded Liposomes in Thermoreversible Gels for Atopic Dermatitis”. In this article, a pharmaceutical liposome-in-gel platform was developed for paeonol delivery, exhibiting temperature-responsive gelation abilities. Systemic characterizations were performed, including liposome morphology, gelling profile, viscosity, pH, drug loading and release, in vitro and in vivo antioxidant activities, etc. This platform may have promising application aspects in atopic dermatitis management.

## 4. Overview of Contributions to Gel Reviews

In addition to the above interesting articles, three gel reviews are included in this Special Issue. The following is a brief introduction to them:

(1) A review from China, entitled “Utilization of Lyotropic Liquid Crystalline Gels for Chronic Wound Management”. This review summarized and discussed gel-based pharmaceutical platforms suitable for chronic wound management. In particular, the authors argued that lyotropic liquid crystalline gels might be one of the most promising systems. The characteristics of lyotropic liquid crystalline gels were analyzed in detail. Of note, we are thrilled to announce that this review was contributed by the Guest Editor team of this Special Issue.
(2) A review from the USA, entitled “Enhancing Therapeutic Efficacy of Curcumin: Advances in Delivery Systems and Clinical Applications”. This review summarized and discussed various drug delivery platforms suitable for curcumin delivery. In particular, the authors argued that gels might be one of the most promising systems. The composition of the reported curcumin gels was analyzed in detail.
(3) A review from Korea, entitled “Pectin Based Hydrogels for Drug Delivery Applications: A Mini Review”. This (mini) review summarized and discussed representative pectin-based hydrogel pharmaceutical platforms. Interestingly, the pectin extraction methods were analyzed in detail. The authors also suggested that investigators in this field pay attention to the toxicity of such systems.

In summary, this Special Issue involved a number of interesting research articles and reviews that have represented the very recent progress of various aspects of pharmaceutical gels, including nanoscale, mesoscale, microscale, and macroscale gels. The indications for these gels also varied. These gels may offer new insights into the design, preparation, development, and application of pharmaceutical gels, and act as references for future studies. We truly appreciate the efforts of our authors, reviewers, and editors in disseminating invaluable knowledge. It is believed that open science in the research area of pharmaceutical gels has advanced, aligning well with the aims of MDPI Press.

## Data Availability

No new data were created in this Special Issue.
